# The Relationship Between Regulatory Emotional Self-Efficacy and Core Self-Evaluation of College Students: The Mediation Effects of Suicidal Attitude

**DOI:** 10.3389/fpsyg.2018.00598

**Published:** 2018-04-24

**Authors:** Xiaojun Zhao, Changxiu Shi

**Affiliations:** ^1^School of Education, Hebei University, Baoding, China; ^2^School of Criminal Justice, China University of Political Science and Law, Beijing, China

**Keywords:** regulatory emotional self-efficacy, suicidal attitude, core self-evaluation, mediation effect, sense of happiness

## Abstract

This study analyzed the mediation effect of a suicidal attitude from regulatory emotional self-efficacy to core self-evaluation. A measurement study was conducted among 438 college students using the Regulatory Emotional Self-Efficacy Scale, the Core Self-Evaluation Scale, and the Suicide Attitude Questionnaire. Results from the plug-in process in SPSS and the bootstrap method showed that the attitude toward suicidal behavior and the attitude toward family members of an individual who has committed suicide played a double-mediation role, from perceived self-efficacy in managing happiness to core self-evaluation. The results also showed that the attitude toward a person who committed suicide or attempted suicide played a mediation effect from perceived self-efficacy in managing curiousness to core self-evaluation. This research has great significance for improving the understanding of college students’ sense of happiness and prevention for self-evaluation.

## Introduction

### Suicide College Students

Suicide in college students is one of the most important aspects of mental health education for college students. Suicidal ideation in college students can be discussed with respect to the following two aspects: college students’ suicide behavior and suicidal attitude. However, suicidal attitude is a more important psychological factor. On the one hand, in social environments, each individual may have suicidal attitude under the influence of various negative stimulations and frustrations. Suicidal ideation occurs primarily at the cognitive level for most people and is not the leading cause of suicidal behavior. On the other hand, suicidal attitude may include the attitude of an individual that is inclined to commit suicide or the attitude of an individual that is not inclined to commit suicide. However, suicidal attitude also includes attitudes toward people who commit suicide, people who are in the environment around the individual, and related social attributes Because family and friends are also important factors affecting a suicide attempt (Frey et al., 2016).

Suicide in college students is due to academic anxiety, interpersonal difficulties, physical health, and other factors (Seiden, 1966). Body image can predict depression, depression can predict alcohol use, and alcohol use can predict proneness to suicide (Lamis et al., 2010). The Anderson–Darling test is an effective tool to identify suicide clustering (MacKenzie, 2013). However, we are more concerned about the significance of college students’ suicidal attitude, rather than the suicide itself. Compared with the intervention of college students’ suicidal behavior, the improvement of college students’ suicide attitude has a more positive, early preventive effect. The studies on college students’ suicidal attitude are important not only for suicide but also for healthy college students.

College students have permissive attitudes about suicidal behavior (Li and Phillips, 2010). After adjusting for reflection and hopelessness, brooding and suicidal ideation are closely linked (Cheref et al., 2015). An ideator with poor problem solving holds a certain attitude about suicide behavior (McAuliffe et al., 2003).

### Perceived Self-Efficacy in Managing Happiness, Suicidal Attitude, and Core Self-Evaluation

Perceived self-efficacy in managing happiness refers to individual efficiency in coping with success or positive events embodied in positive emotions (Zhao et al., 2013). Related studies found that psychological elastic impact core self-evaluation (Zeng et al., 2014). Studies have found that core self-evaluation at a high level and emotion regulation ability at a high level were closely related (Kacmar et al., 2009). In addition, Judge thinks that self-efficacy affects core self-evaluation (Judge and Hurst, 2007). As mentioned earlier, perceived self-efficacy in managing happiness was a combination of the effects of self-efficacy and optimism. Therefore, perceived self-efficacy in managing happiness may affect core self-evaluation. The study put forward H1 as follows: the perceived self-efficacy in managing the happiness of college students has a significant effect on core self-evaluation.

Do suicidal attitudes, which are important in relation to people’s outlook on life, play an indirect role for perceived self-efficacy in managing happiness? Suicide is the third largest factor of death for college students. Fear/anxiety and suicide attempts or self-injury are closely related (Mitchell et al., 2014). Depression significantly affects suicidal ideation (Westefeld and Furr, 1987; Furr et al., 2001; Mackenzie et al., 2011). At the same time, perceived burdensomeness can predict suicidal ideation (Wong et al., 2011). Fear/anxiety, depression, and burdensomeness belong to the category of mood. However, self-efficacy indirectly influences suicidal ideation through optimism (You and Chen, 2012). Optimism leads to low depressive symptoms or hopelessness and suicidal ideation (Bryan et al., 2013; Chang et al., 2017). In addition, there are studies that make it clear that optimism can reduce suicide risk for college students (Hirsch et al., 2007). From the perspective of psychological experience, does perceived self-efficacy in managing happiness with a combination of self-efficacy and optimism affect suicidal attitude? Suicidal ideation is partly affected by individual emotional distress, and regulatory emotional self-efficacy serves as an important aspect of emotional control related to the effects of individual suicide or attitude. Normally, the mental flexibility of an individual with a suicide identity is poor.

As a carrier of the characteristics, suicidal attitude may affect a person’s overall core self-evaluation. The suicide attitude connected with one’s life outlook is involved in the philosophy of life. The suicide attitude has an important influence on one’s overall self-analysis. In a study of suicidal attitude, the researchers focused on attitudes toward suicide. At the same time, the family caregivers (such as family members of suicide) are an important link to reduce the number of suicides (Chiang et al., 2015). The study put forward H2 and H3 as follows. H2: the perceived self-efficacy in managing the happiness of college students indirectly affects core self-evaluation through the attitude for suicidal behavior; H3: the perceived self-efficacy in managing the happiness of college students indirectly affects core self-evaluation through attitude to family members of suicide.

### Perceived Self-Efficacy in Managing Curiousness and Suicidal Attitude

Curiosity and anxiety can be regarded as two kinds of reverse drive and emotional state. The interaction of curiosity and anxiety affect the individual’s attitude and behavior (Spielberger and Starr, 1994). Particularly, interpersonal curiosity is better. For an individual of high interpersonal curiosity, it is easier to understand social behavior. In social activities, an individual showing quality curiosity has more positive emotions, creating a more positive and close social experience (Kashdan et al., 2011). An individual with poor control over interpersonal curiosity may eventually exhibit deviant behavior. So, from the perspective of interpersonal environment, perceived self-efficacy in managing curiousness may affect suicidal attitude, particularly the suicidal attitude toward the person who committed suicide or attempted suicide. The study put forward H4 as follows: the perceived self-efficacy in managing the curiosity of college students indirectly affects core self-evaluation through the suicidal attitude toward a person who committed suicide or attempted suicide.

Some people have affirmatory suicidal attitude, but few people display suicidal behavior. An attitude toward suicide might affect the person’s overall perception and self-evaluation. Happiness is one of the important indicators of modern social development. This study has important significance for improving the understanding of college students’ sense of happiness and prevention for self-evaluation.

## Materials and Methods

### Participants

The participants included 450 university students from Anhui province and Hebei province. All participating universities had cases of suicide or attempted suicide in the past year. The participants completed the informed consent form and the basic information form. The study was approved by the academic and ethics committee of school of education in Hebei University. The number of students who completed the recycling questionnaire was 441, and the recovery rate was 97.33%. After excluding invalid questionnaires, the number of participants was 438, and the effective rate was 99.32%. The participants included 128 boys and 310 girls, and 124 student cadres and 314 non-student cadres.

### Measures

#### Regulatory Emotional Self-Efficacy (RESE) Scale

We used the RESE Scale of Chinese college students (Zhao et al., 2013). This scale included 42 items, using a 5-point scoring method. The RESE scale of Chinese university students consists of seven factors, including perceived self-efficacy in managing inferiority, happiness, jealousy, horror/fear, confidence, curiosity, and reliance. The total internal consistency coefficient is 0.913 in this test.

#### Suicide Attitude Questionnaire

This study uses the Suicide Attitude Questionnaire (Xiao et al., 1999). This scale includes 29 items and uses a 5-point scoring method. The scale is divided into four factors, namely, attitude toward suicide behavior (F1), attitude toward a person who committed suicide or attempted suicide (F2), attitude toward family members of an individual who has committed suicide (F3), and attitude toward euthanasia (F4). The total internal consistency coefficient is 0.647 in this test.

#### Core Self-Evaluation Scale

This study used the Chinese version of the Revised Core Self-Evaluation Scale (Du et al., 2012). This scale included 10 items and used a 5-point scoring method. The scale is only one factor. The internal consistency coefficient is 0.719 for this scale.

### Process

The study used paper and pencil tests. The experimenters were psychology teachers with Master’s or Doctorate degrees. The participants (college students) took part in psychological tests with unified instructions. The psychological tests included the following: the RESE Scale, the Suicide Attitude Questionnaire, and the Core Self-Evaluation Scale. During the self-study period of the college students’, college students filled out these questionnaires. Then, the completed questionnaires were scored and analyzed.

### Data Management and Analysis

The study conducted data management and analysis using the PROCESS plug-in of the SPSS 17.0 software (Hayes, 2013). The study carried out a correlation analysis and mediation effects analysis.

## Results and Analysis

### Correlation Analysis

According to the results of the correlation coefficient (Pearson) among perceived self-efficacy in managing happiness, perceived self-efficacy in managing curiousness, attitude toward suicidal behavior, attitude toward family members of suicide, attitude toward a person who committed suicide or attempted suicide and core self-evaluation (see **Table 1**), there were two significant correlations among perceived self-efficacy in managing happiness, attitude toward suicidal behavior and core self-evaluation. There were two significant correlations among self-efficacy in managing happiness, attitude toward family members of an individual who has committed suicide and core self-evaluation. The correlation between perceived self-efficacy in managing curiousness and attitude toward a person who committed suicide or attempted suicide was significant (*p* < 0.01). The correlation between the attitude toward a person who committed suicide or attempted suicide and core self-evaluation was significant (*p* < 0.001). The variables involved in the study are significantly correlated. To further verify the hypothesis, the study conducted the corresponding mediation effect analysis.

**Table 1:** The correlation based on perceived self-efficacy in managing happiness and curiousness.

	*M* ± *SD*	1	2	3	4	5	6
(1) Perceived self-efficacy in managing happiness	4.230 ± 0.466	1					
(2) Perceived self-efficacy in managing happiness	3.290 ± 0.763	0.295^∗∗∗^	1				
(3) Attitude for suicidal behavior	3.070 ± 0.570	-0.134^∗∗^	0.029	1			
(4) Attitude toward a person who committed suicide or attempted suicide	2.700 ± 0.424	-0.070	0.128^∗∗^	0.072	1		
(5) Attitude toward family members of an individual who has committed suicide	2.504 ± 0.450	-0.236^∗∗∗^	0.018	-0.039	0.404^∗∗∗^	1	
(6) Core self-evaluation	3.256 ± 0.512	0.182^∗∗∗^	0.000	0.390^∗∗∗^	-0.222^∗∗∗^	0.156^∗∗∗^	1

*^∗^p < 0.05, ^∗∗^p < 0.01, ^∗∗∗^p < 0.001.*

### Double Mediation Effect Analysis Based on Perceived Self-Efficacy in Managing Happiness

**FIGURE 1:**
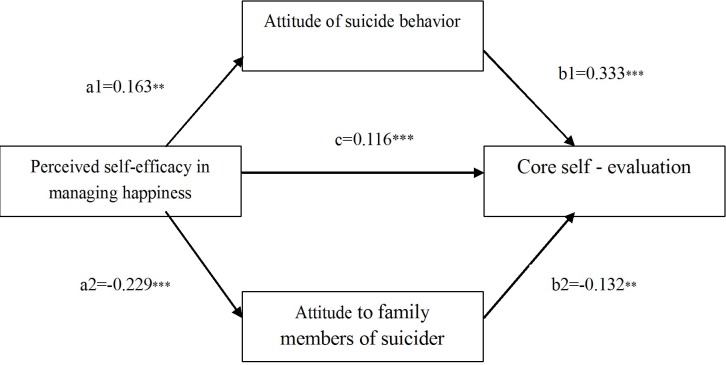
Mediation effect diagram for attitude of suicide behavior and attitude toward family members of an individual who has committed suicide.

To further analyze the relations among perceived self-efficacy in managing happiness, attitude for suicidal behavior, attitude toward family members of an individual who has committed suicide and core self-evaluation, the study conducted a mediation effect analysis. The analysis diagram of the mediation effect is shown in **Figure 1**.

The study conducted a mediation effect analysis using the process proposed by Zhao et al. (2010), which utilized the Bootstrap method proposed by Preacher and Hayes (2004) and Hayes (2013). Data analysis was done using the PROCESS plug-in of SPSS (Hayes, 2013). The sample size of the Bootstrap analysis was 5000. Under the 95% confidence interval, the mediation effect test results do not contain 0. The direct effect of A on B was 0.116 (LLCI = 0.018, ULCI = 0.213). The indirect effect of A on B was 0.085 (LLCI = 0.038, ULCI = 0.137). Based on F1, the indirect effect of A on B was 0.054 (LLCI = 0.015, ULCI = 0.100). Based on F3, the indirect effect of A on B was 0.030 (LLCI = 0.007, ULCI = 0.066). There was a partial mediation effect found. The results showed that the double mediation effects of attitude toward suicide behavior and attitude toward family members of an individual who has committed suicide were significant. However, the mediating effect is a complementary mediating effect. Therefore, future research should consider whether there are other intermediary variables. The process from perceived self-efficacy in managing happiness to core self-evaluation included the direct and indirect effects of attitude toward suicide behavior and attitude toward family members of an individual who has committed suicide as double mediation.

### Mediation Effect Analysis Based on Perceived Self-Efficacy in Managing Curiousness

**FIGURE 2:**
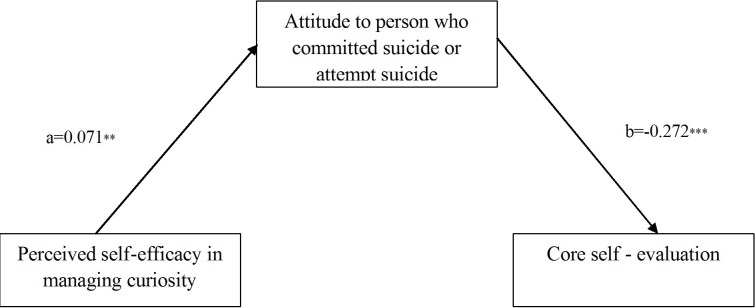
Mediation effect diagram for attitude toward a person who committed suicide or attempted suicide.

To further analyze the relations among perceived self-efficacy in managing curiousness, attitude toward a person who committed suicide or attempted suicide and core self-evaluation, the study conducted a mediation effect analysis. The analysis diagram of the mediation effect is shown in **Figure 2**.

The study conducted a mediation effect analysis program. The data analysis used the PROCESS plug-in of SPSS (Hayes, 2013). The sample size of the Bootstrap analysis was 5000. Under the 95% confidence interval, the mediation effect test results do not contain 0. The indirect effect of C on D was -0.019 (LLCI = -0.042, ULCI = -0.006). The direct effect of C on D was 0.019 (LLCI = -0.043, ULCI = 0.081); therefore, a complete mediation effect was found. The results showed a significant mediation effect of the attitude toward a person who committed suicide or attempted suicide.

## Discussion

### Perceived Self-Efficacy in Managing Happiness Impact on Core Self-Evaluation

The study confirms H1, and the results are in line with previous studies (Judge and Hurst, 2007; Kacmar et al., 2009; Zeng et al., 2014; Zang et al., 2015). Psychological elastic, emotion regulation ability, and self-efficacy affect core self-evaluation. In this study, perceived self-efficacy in managing happiness refers to individual efficiency in coping with success or positive events embodied in positive emotions (Zhao et al., 2013). The core self-evaluation is a basic evaluation for their ability and value (Judge et al., 1997). Emotional elasticity is a type of regulation ability of negative emotion, and perceived self-efficacy in managing happiness reflects your perceptive ability to adjust to a happy mood in a controlled manner. These two aspects are associated in that perceived self-efficacy in managing happiness is a factor of regulatory emotional self-efficacy, as well as a type of special self-efficacy. This special self-efficacy is also likely to affect the general integrity of self-evaluation. Perceived self-efficacy in managing happiness with a nature of optimism is a type of positive individual ability. This ability will become a personality trait if it is positively exercised for a long time. This trait ultimately leads to increased ability and value.

### Intermediary Effect Analysis of the Suicidal Attitude

Perceived self-efficacy in managing happiness is a reflection of positive psychology. Perceived self-efficacy in managing happiness of the optimistic nature is bound to influence one’s perceptions (negative or positive) of suicide. Due to differences in cognitive nature, attitude led to the observed differentiation of the college students’ self-evaluation.

Previous studies have found that self-efficacy indirectly influences suicidal ideation through optimism (You and Chen, 2012). Optimism reduces depressive symptoms (Hirsch et al., 2014), depression significantly affects suicidal ideation (Westefeld and Furr, 1987; Furr et al., 2001; Mackenzie et al., 2011). Depression belongs to the category of emotional symptoms, but optimism is a rather positive psychology. If the individuals have the ability to adjust the level and perception of their happiness, then the suicidal attitude may be improved. Once one has the ability to control environmental factors, the individual will reduce negative thoughts and actions. In contrast, individual feelings that are passively controlled by the environment will produce more negative thoughts and actions. A suicidal attitude also includes attitudes toward suicidal behavior, people who commit suicide, people who are in the environment around them, and related social attributes, since family and friends are also important factors affecting a suicide attempt (Frey et al., 2016). The study found that attitudes toward suicidal behavior and attitudes toward family members of a suicide were mediated. The perceived self-efficacy in managing happiness with a combination of self-efficacy and optimism affects suicidal ideation.

The perceived self-efficacy in managing curiousness reflects the adjustment ability of interpersonal curiosity. For an individual of high interpersonal curiosity, it is easier to understand social behavior. In social activities, an individual with quality curiosity has more positive emotions, thus creating a more positive and close social experience (Kashdan et al., 2011). A suicidal attitude is a mixture of emotion and social experience. Negative emotion and negative social experience a prompted more negative suicidal attitude. A college student with poor control over interpersonal curiosity may eventually exhibit deviant behavior. These deviant behaviors include suicide behavior and internal fighting behavior.

Related research showed that the core self-evaluation influenced suicidal ideation (Ma et al., 2013). However, this study considered that a suicidal attitude affects the core self-evaluation for the following reasons: (1) suicidal ideation and attitude for suicidal behavior are different. Suicidal ideation refers to oneself, and attitude for suicidal behavior refers to oneself or others; (2) whether related to oneself suicide or suicide involved with others, the individual’s outlook on life is severely affected if one has a positive attitude toward suicide. The individual’s view of life promotes a change in the self-assessment.

### Prevention for Core Self-Evaluation Based on Perceived Self-Efficacy in Managing Happiness and Suicidal Attitude

The traditional intervention of core self-evaluation rarely considers the perceived self-efficacy in managing happiness and suicidal attitude. Positive psychological constructs should be an important factor in suicide intervention (Celano et al., 2017). Intervention for suicidal attitudes may be more likely to lead to a deeper variation in self-evaluation. Because suicide does not occur in a majority of people, most healthy people have a stable attitude toward suicide. A stable attitude toward suicide may have a stronger effect on one’s outlook on life. The intervention plan with some techniques for building scenarios (such as virtual reality and augmented reality) may have profound implications for the ascension of self-evaluation. In addition, perceived self-efficacy in managing happiness was a combination of self-efficacy and optimism. Perceived self-efficacy in managing happiness directly affects one’s attitude toward suicide and directly influences core self-evaluation. It is a reliable method to improve one’s perceived self-efficacy in managing happiness through behavioral training techniques.

### Limitations and Future Directions

Although this study confirmed the mediation effect of a suicidal attitude on perceived self-efficacy in managing happiness/curiousness to core self-evaluation, the following limitations were present: (1) the sample size could be larger; (2) only a single research method is used; (3) because the double-mediation effect in this study is a complementary mediating effect, there may not be any other intermediary variables. Future studies should aim to do the following: (1) analyze the mediation effects among core self-evaluation, attitude for suicidal behavior, and suicidal behavior by confirming whether there is a chain mediation effect; (2) confirm the related mechanism of sense of happiness using susceptibility theory; determining whether there is a susceptibility factor for sense of happiness would be of great value.

## Author Contributions

XZ and CS conceived and designed the study, performed the study, analyzed the data, and wrote the paper.

## Conflict of Interest Statement

The authors declare that the research was conducted in the absence of any commercial or financial relationships that could be construed as a potential conflict of interest.
